# Revisiting the fear of snakes in children: the role of aposematic signalling

**DOI:** 10.1038/srep37619

**Published:** 2016-11-25

**Authors:** Jérémie Souchet, Fabien Aubret

**Affiliations:** 1 Station d’Ecologie Théorique et Expérimentale, CNRS, 09200 Moulis, France

## Abstract

Why humans fear snakes is an old, yet unresolved debate. Its innate origin from evolutionary causes is debated against the powerful influence early experience, culture, media and religion may have on people’s aversion to snakes. Here we show that the aversion to snakes in human beings may have been mistaken for an aversion to aposematic signals that are commonly displayed by snakes. A total of 635 children were asked to rate single item images as “nice” or “mean”. Snakes, pets and smiley emoticon items were not rated as “mean” unless they displayed subtle aposematic signals in the form of triangular (rather than round) shapes. Another 722 children were shown images featuring two items and asked which item was “nice” and which item was “mean”. This context dependent comparison triggered even sharper responses to aposematic signals. We hypothesise that early primates evolved an aversion for aposematic signals in the form of potentially harmful triangular shapes such as teeth, claws or spikes, not for snakes per se. Further, we hypothesise that this adaptation was in turn exploited by snakes in their anti-predatory threat display as a triangular head or dorsal zig-zag pattern, and is currently the basis for efficient international road-danger signalling.

Snakes are one of the most common subjects of intense fears and phobias in humans^1^,^2^,^3^,^4^,^5^. Fearful reactions to snakes have also been reported for a variety of nonhuman primates^4^,^6^,^7^. Some authors suggested that an evolutionary arms race between early constrictor snakes and mammals triggered the development of orbital convergence, visual specialisation, and brain expansion in early primates; and incidentally the current human reactions observed in response to the appearance of venomous snakes (viperids and elapids^3^,^4^,^8^). The belief that humans have an innate fear of snakes^9^,^10^ was recently supplanted by the more refined idea that humans may have inherited an evolved tendency to associate snakes (or certain snake features such as the slithering motion^11^) with fear^4^,^12^,^13^. The capacity to define and quickly identify a threat signal would indeed provide a clear selective advantage. Hence, the tendency of some individuals to be afraid of threat signals would have evolved in mammals, creating an innate disposition to the acquisition of this fear^11^,^14^. Yet the dislike, or fear of snakes may be also an acquired taste: while babies and very young children do not usually fear snakes, they are unusually skilled at detecting them and show a predisposition to learn to fear snakes if they have bad experiences or even if they are exposed to negative portrayals of them in the media^14^. Sharp fear-relevant responses to snake stimuli such as heart rate acceleration were recorded in conditioned adult humans^13^.

Despite the seemingly strong idea of an evolutionary arms race between early constrictor snakes and mammals, human beings are not natural prey for snakes. Even to the largest species of pythons, early humanoids such as *Australopithecus* were probably too large a prey. On the other hand venomous snakes such as vipers, mostly small and cryptic ambush predators with potent venom, were most likely as abundant in Africa as they are today^15^. Detecting and avoiding venomous snakes may have contributed to survival of early humans and primates. While ambush predators such as vipers need to be invisible to their prey, they have evolved a number of traits to either deter potential predators or to avoid being trampled, such as warning or aposematic signals. Warning signals may be (or combine) patterns, colours, shape, behaviour, odours and sounds^16^,^17^. Harmful snakes and their mimics boast aposematic signals such as bright colours^18^, rattle or hissing sounds^19^,^20^, dorsal zigzag patterns^21^,^22^ and shaping into or accentuating the triangular shape of their head when threatened^23^. It was shown that plasticine models of snakes displaying a dorsal zig-zag pattern or a triangular head shape are less likely to be attacked by predators^21^,^22^. There is also some experimental evidence that predators can recognise the typical triangular head shape of vipers used as a warning signal^23^.

How human beings detect and assess aposematic signals has, to our knowledge, not been explored and could hold the key to our current (mis)understanding of snake fear and phobia. We tested school children in Southern France (see map - S1) for their response to snake and non-snake stimuli (Fig. 1), with or without aposematic signals under a balanced rigorous protocol in order to tease apart the influence of age, sex, and stimulus. Tests were designed to assess the raw perception of children of a single item image (Fig. 1A to D) as either nice or mean (listen to S2 single item image); and of double item images (Fig. 1E to H) in order to trigger a context-dependant response (i.e. children were asked to indicate which of the two items was nice and which was mean – listen to S2 double item image).

## Results

### Single item images (N = 635 children)

A General Linear Model (Table 1) was used to test the relative influences of age, sex, adjective order and image type (factors) on the scoring (nice or mean; dependent variable). Image type had a significant influence on the test outcome (see Fig. 2). There was a tendency for children age to significantly influence the outcome: 3 years old children were more inclined to label items as nice while 7 years old children more inclined to label things as mean (see Fig. 3). Unsurprisingly, children predominantly identified the rabbit and dog items (A1 and A2) as being respectively nice and mean (only three young children gave the opposite response to that expected). All snake drawings (with or without aposematic signal; B1 to B4) were overall perceived as mean. The cumulative effect of two aposematic signals (B4) over just one (B2 or B3) did not trigger a stronger response. The photograph of a snake head (C1) generated a neutral response, but the same snake head displaying a threat signal (triangular head – C2) was predominantly assigned as mean. Finally, while emoticon smileys featuring round teeth were clearly of nice appearance (D1), triangular teethed emoticon smileys (D2) appeared as mean to a majority of children.

### Double item images (N = 722 children)

The results obtained with a context dependant interpretation reinforced the trends observed with single item images (see Fig. 4). In all double item images tests, items featuring one aposematic signal were primarily designated as the mean items. Further, the presence of two aposematic signals also elicited the mean designation alongside the presence of only one signal (B4 *vs* B3).

#### The recognition of aposematic signals

In addition to single and double item image results, we aimed at assessing the children’s ability to identify aposematic signals in an *a priori* “correct” way (i.e. a threatening dog is mean, a rabbit is not) and the respective influence of sex, age, adjective order and context (single or double item images) on the outcome. We ran a General Linear Model with children age, sex, adjective order and item types as nested categorical factors into image category (single or double items). Age, image type and image category had a significant effect on the outcome (Table 2). The percentage of correct identification of the aposematic signals increased from 3 to 6 years old in both single and double item images. Correct identification tended to plateau in older children tested with double item images, while remaining unpredictable with single item images (Fig. 5); highlighting the key importance of context (i.e. comparison).

## Discussion

Our tests with single and double item images demonstrated that the decision process in the vast majority of children was influenced by the presence of aposematic signalling: snakes, pets and smiley emoticon images were overall rated as “nice” unless they displayed one or two subtle aposematic signals in the form of triangular (rather than round) shapes (triangular head, zig-zag pattern or sharp teeth). Overall, children of all ages were strikingly skilled at identifying the aposematic signals, especially when provided double item images (Figs 4 and 5). Tests using double item images also highlighted the importance of context (comparison) in the ability of children of all ages to correctly identify aposematic signals and even rank them (two aposematic signals were more influential than just one). Younger children (3 to 6 years old) were less skilled at either identifying aposematic signals or alternatively less inclined to interpret them as “danger” signalling. This suggests that the recognition and interpretation of aposematic signalling may benefit from experience and/or reflect some level of brain immaturity in younger children in this regard.

While we provided a direct image for comparison (i.e. no aposematic signal *versus* one), it is important to note that a mental comparison may also occur in reference to any prior experience a child may have undergone, in the form of direct encounters (i.e. first-hand experience with an aggressive barking dog for instance), media portrayal, word of mouth (from friends, parents) or even religiously carried representations. This is especially relevant to snake related fears as snakes are systematically portrayed in modern societies as dangerous, harmful, unpleasant or symbols of evil. That is, the actual seeing of a snake (in the wild, on television or at the zoo) may be immediately referenced against what one has heard, read or seen about snakes. While it has been suggested that the fear of snakes stemmed from an early mammal anti-predatory response, we suggest that, instead, or in addition to, early mammals evolved an aversion to shapes and things that could potentially harm them. The latter may include sharp teeth, claws, angular rocks (where careful treading is usually observed in mammals), as well as vegetal and animal spikes and horns. Knives, scissors and needles may nowadays also fall into this category. Further, it is often believed that snakes flatten their head in a triangular shape to appear more formidable and deter potential predators. Yet, viper mimics clearly flatten their head to deceive predators by appearing as a true viper, not necessarily as a large snake^23^,^20^. Hence, a possibility exists that snakes have exploited the recognition by mammals (including humans) and birds^21^ of danger signalling: most snakes (harmless or venomous) adopt a triangular head pose when threatened because the shape is perceived as potential danger by predators (i.e. aposematic signalling) or any large animals that pose a trampling threat. On a different note, it is equally striking that this mechanism has been exploited by human beings themselves in modern societies. Cartoon characters are an effective example: nice friendly “good” characters are usually drawn with round edges, while their rival “evil” character often displays sharp angles and triangular teeth (i.e. Mufasa *versus* Scar in The Lion King^®^; Lenny *versus* Don Lino in Shark Tale^®^). Finally, a direct extension of the human awareness of aposematic signals can be seen in the form of danger signalling in road signing^24^, workplace health and safety and most general safety signage. A Google^®^ image search for the words “danger + signal” highlights the ubiquity of brightly coloured triangular signalling. We believe that the association between shape and colour in danger signalling is extremely informative: frugivory in primates is thought to have favoured the visual specialisation of trichromatic colour vision in order to detect red and orange fruits against a background of green foliage as well as to efficiently detect fruit quality and ripeness^25^,^26^,^27^. It is tempting to advocate for a comparable adaptation for shape recognition of sharp triangular aposematic signals. In fact, various asymmetries in visual searches were established in human beings. For instance, a curved target among rectilinear stimuli is more easily detected than a rectilinear target among curves^28^. Further, mammals share common structures of the brain that are involved in vigilance, fear, and learning associated with fearful stimuli. Some of these structures, such as the koniocellular visual pathway and the parvocellular pathway, have expanded in primates and are strongly involved with colour and object recognition (i.e. the mammalian fear module^4^,^8^).

In conclusion, we propose that early primates evolved an aversion for aposematic signals in the form of potentially harmful triangular shapes that are commonly displayed by snakes, not for snakes *per se*. Our study provides unprecedented insights about the perceptual mechanisms and associated visual features in relation to aposematic signals that give snake stimuli privileged access to the mammalian fear module^4^,^12^,^13^.

## Methods

A total of 1357 children were tested, including 685 boys (mean age = 6.91 ± 2.21) and 672 girls (mean age = 6.92 ± 2.24), aged from 3 to 11 years old (median age = 7.0). The distribution of sexes across age classes was not significantly skewed (χ^2^ = 9.18; df = 8; P = 0.33). Thirty-two primary schools were visited in the region Midi-Pyrénées, France (Figure SM1) from January to May 2015. All experiments were performed in accordance with relevant guidelines and regulations (Ministère de l’Education Nationale). The methods were carried out in accordance with the relevant guidelines (Circulaire n° 92.196 du 3 juillet 1992). All experimental protocols were approved by the Académie de Toulouse (Education Nationale Midi-Pyrénnées). Informed consent was obtained from all subjects and their legal representatives (parents and heads of schools). Each school was visited only once and each child tested only once. Under current French laws (Circulaire n° 92.196 du 3 juillet 1992), no ethics approval was required for this study.

**Figure 1:**
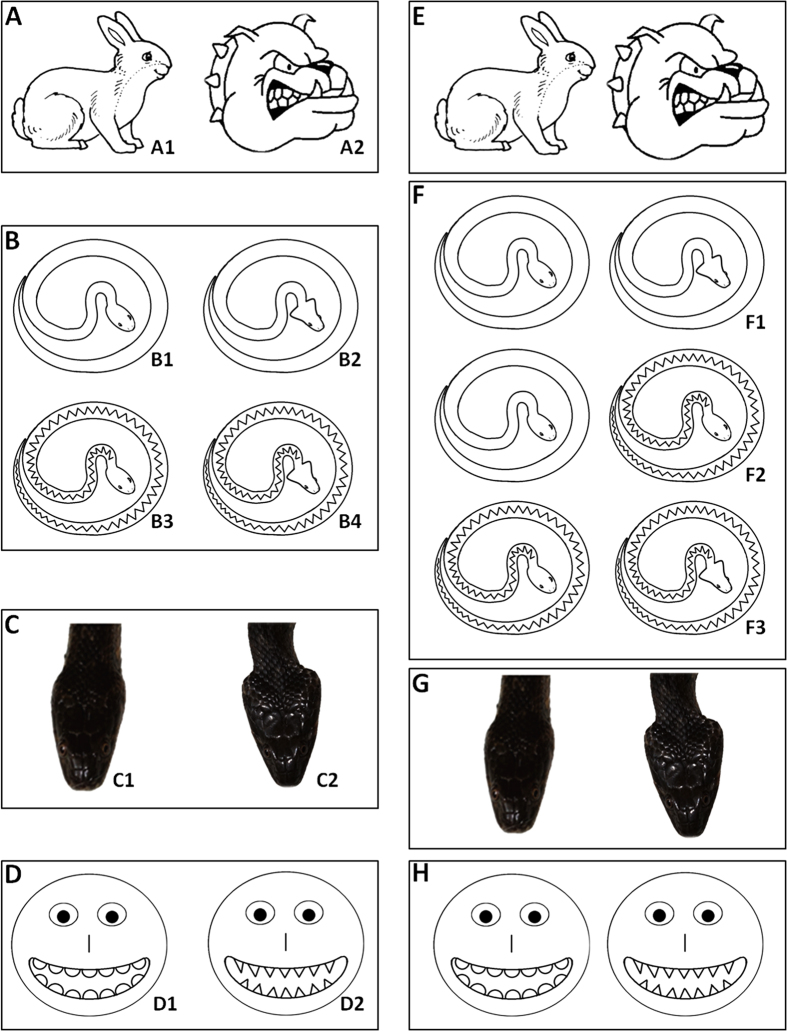
We used 10 single item images and 10 double item images to assess the perception of aposematic signalling in children. Each child was only tested once. In the case of single item images, children (N = 635) were asked to assess the item as representing something “nice” or “mean”. Categories included: (**A**) control items with a familiar picture of a rabbit (A1) and an aggressive dog (A2); (**B**) snake drawings featuring a control snake (B1), snake with triangular head (B2; one aposematic signal), snake with dorsal zig-zag (B3; one aposematic signal), and a snake with both a triangular head and dorsal zig-zag (B4; two aposematic signals); (**C**) head photograph of a harmless viperine water snake (*Natrix maura*; mimic of European adders *Vipera sp*.) in normal (C1, round head) and defensive mimicry (C2, triangular head) mode; and (**D**) emoticon smileys featuring round (D1) or triangular (D2) teeth. Tests using double item images (**E** to **H**) were used to assess context dependent interpretation of aposematic signalling in children (N = 722).

**Figure 2:**
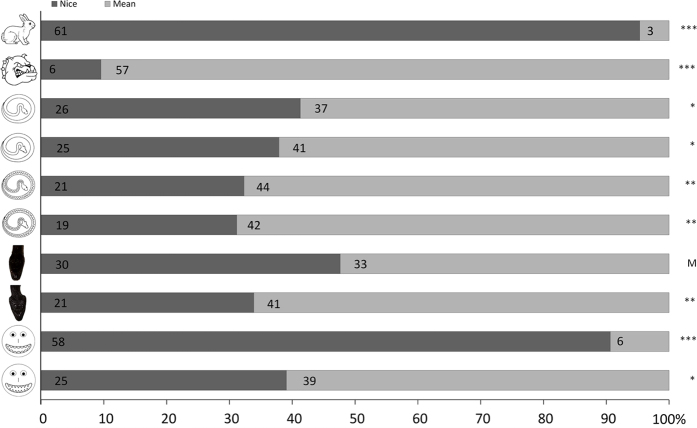
Results of single item image tests in school children. Ns are indicated in the horizontal bars along with the results of Binomial tests for each test. Probability values follow the coding: ***P < 0.001; **0.001 < P < 0.01; *0.01 < P < 0.05; M 0.05 < P < 0.10; NS P > 0.10.

**Figure 3:**
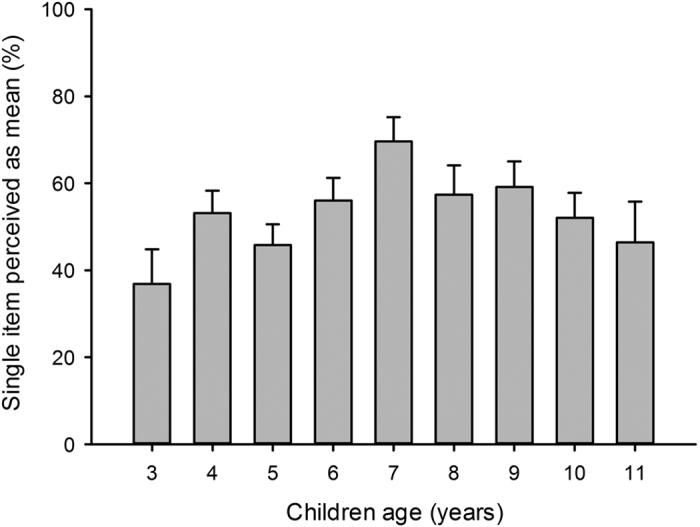
Proportions of single item images perceived as mean by children aged 3 to 11 (N = 635). Mean ± SE are plotted.

**Figure 4:**
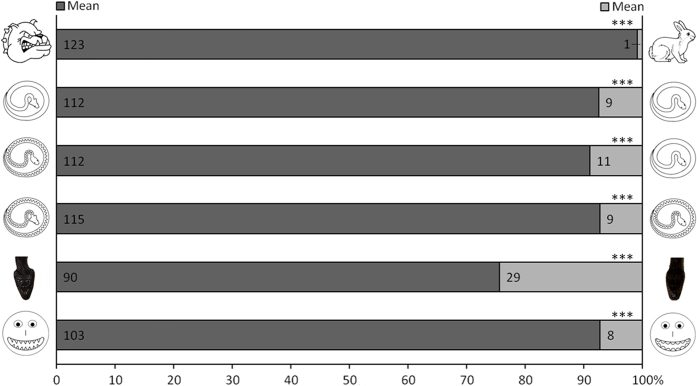
Results of double item image tests in schoolchildren. Ns are indicated in the horizontal bars and refer to the number of children that designated each item as mean (and by default the alternate item as nice). The results of binomial tests for each test (ages pooled) are given (***P < 0.001; **0.001 < P < 0.01; *0.01 < P < 0.05; M 0.05 < P < 0.10; NS P > 0.10).

**Figure 5:**
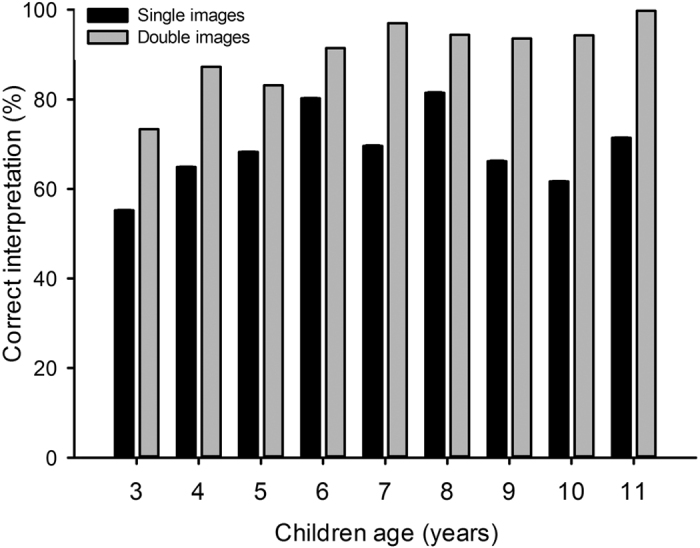
A General Linear Model was run with age, sex, adjective order and item type as Nested categorical factors into image category (single or double). The effect of age on the correct identification of aposematic signals in schoolchildren in the case of single and double item images is plotted (F_16, 1321_ = 2.76, P < 0.001). Very young children “incorrectly” identified aposematic signals more often than older children in double item images, but not in single item images. The graph highlights the importance of context in aposematic signal interpretation in children of all ages: percentages of correct identification of aposematic signals were consistently higher with double item images than with single item images.

Two categories of tests were carried out (Fig. 1): single item image tests (N = 10) and double item images tests (N = 6). Testing used a balanced order design^29^ and each child was only tested once. Items (drawings or pictures; Fig. 1) were standardised in size, colour (black and white) and orientation (head orientated to the right, where relevant). To avoid potential confounding factors, all tests were performed by one person (J. S.). Children were asked to listen to one question (digital voice formulated using the freeware Dys-Vocal^®^) through earphones. The order of the adjectives used (“mean” or “nice” – see below) was alternated^30^.

The questions used child-friendly wording in French and can be accurately translated to English (original audio files are available as SM2) as:

- Single item images: “*Have a look at this picture and tell me if what you see is something nice or something mean” and “Have a look at this picture and tell me if what you see is something mean or something nice*”.
- Double item images: “*Have a look at these pictures and tell me which one is nice and which one is mean” and “Have a look at these pictures and tell me which one is mean and which one is nice*”.

## Additional Information

**How to cite this article**: Souchet, J. and Aubret, F. Revisiting the fear of snakes in children: the role of aposematic signalling. *Sci. Rep.* **6**, 37619; doi: 10.1038/srep37619 (2016).

**Publisher's note:** Springer Nature remains neutral with regard to jurisdictional claims in published maps and institutional affiliations.

## Supplementary Material

Supplementary Information

Supplementary S2 Single Item Image A

Supplementary S2 Single Item Image B

Supplementary S2 Double Item Image A

Supplementary S2 Double Item Image B

## Figures and Tables

**Table 1:** A total of 635 schoolchildren were individually asked to assess images shown on a screen as being nice or mean (see Fig. 1).

	*Dl*; *F*	*P*
Age	1; 3.16	0.076 (M)
Sex	1; 0.51	0.47 (NS)
Image type	9; 24.59	0.001 (***)
Adjective order	1; 0.01	0.91 (NS)

A General Linear Model was run with test results (nice or mean) as dependent variable, age (3 to 11 years old) as continuous predictor, and sex (boy or girl), adjective order in the audio file (mean pronounced first or nice pronounced first) and image type (A1, A2, B1, B2, B3, B4, C1, C2, D1, D2) as categorical factors. Main effects are shown (all interactions were non-significant). Probability values follow the coding: ***P < 0.001; **0.001 < P < 0.01; *0.01 < P < 0.05; M 0.05 < P < 0.10; NS P > 0.10.

**Table 2:** A total of 1357 schoolchildren were individually asked to assess single or double items shown on a screen as being nice or mean (see Fig. 1).

	*Dl*; *F*	*P*
{1} Image category	1; 84.12	0.001 (***)
{2} Sex (within {1})	1; 1.01	0.36 (NS)
{3} Age (within {1})	16; 2.76	0.001 (***)
{4} Adjective order (within {1})	2; 0.14	0.87 (NS)
{5} Image type (within {1})	14; 12.41	0.001 (***)

Images were arbitrarily given a reference depending on the presence or absence of aposematic signals; single item images: 0 or 1 aposematic signal; double item images: 0 versus 1 or 1 versus 2 aposematic signals. We then scored the responses as correct assignment of nice and mean. A General Linear Model was run with age, sex, adjective order and image type as nested categorical factors into item category (single or double). Probability values follow the coding: ***P < 0.001; **0.001 < P < 0.01; *0.01 < P < 0.05; M 0.05 < P < 0.10; NS P > 0.10.
