# Supplementary material for: Revisiting the fear of snakes in children: the role of aposematic signalling
Source: Sci Rep. 2016 Nov 25;6:37619. doi: 10.1038/srep37619 (PMC5122844; doi:10.1038/srep37619)
Supplement: Supplementary Information [file srep37619-s1.pdf]

Online Supplementary Material:

Revisiting the fear of snakes in infants: the role of aposematic signalling

SOUCHET Jérémie & AUBRET Fabien

SM1: Map showing the 32 schools visited during the course of the study, produced using ArcGIS® 10.2.2. (Licence #EFL977377207).

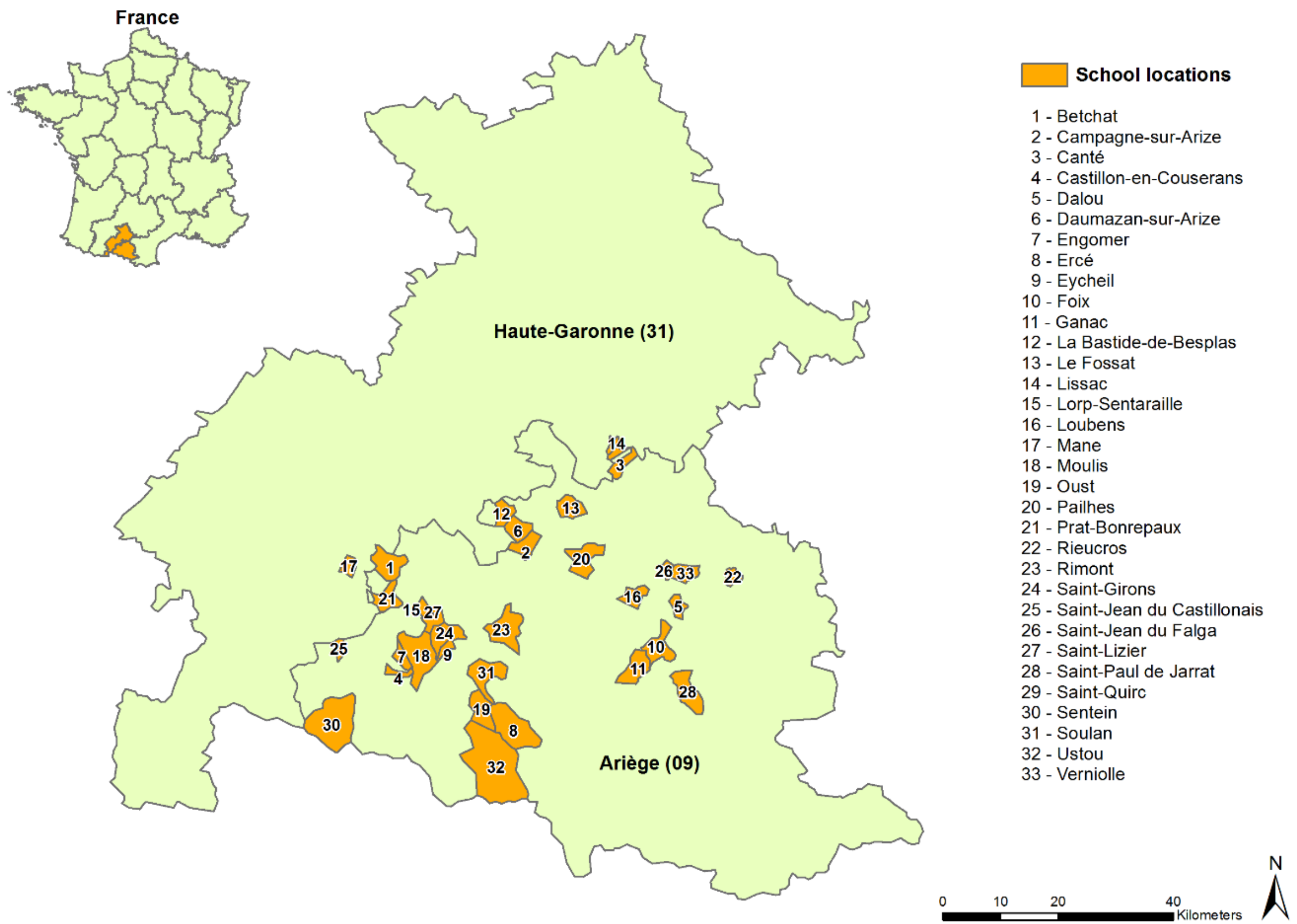

SM2: Audio files used in single (S2 single item image; a and b) and double (S2 double item images; a and b) item tests. We used a balanced design where the order of the adjectives “mean” and “nice” were alternated for each test (a: nice first, mean last; b: mean first, nice last). Single item images audio files can be translated as “*Have a look at this picture and tell me if what you see is something nice or something mean*” and “*Have a look at this picture and tell me if what you see is something mean or something nice*”. Double item images can be translated as “*Have a look at these pictures and tell me which one is nice and which one is mean*” and “*Have a look at these pictures and tell me which one is mean and which one is nice*”. Copyright© Dyslogiciel 2011-2014. All rights reserved.
